# Coli bond: A dual-function encryption system for secure information storage and transmission by microorganisms

**DOI:** 10.1371/journal.pone.0325926

**Published:** 2025-06-11

**Authors:** Xuefeng Xiao, Yunuo Song, Jingxuan Hu, Jinchen Han, Wanbin Xing, Chang Liu

**Affiliations:** 1 Beijing Huijia Private School, Beijing, China; 2 BASIS International School Shenzhen, Shenzhen, China; 3 The Independent Schools Foundation Academy, Hong Kong, China; 4 Wright Schools, Tacoma, United States Of America; 5 Aulin College, Northeast Forestry University, Harbin, China; 6 School of Life Science, Northeast Forestry University, Harbin, China; Shiraz University, IRAN, ISLAMIC REPUBLIC OF

## Abstract

With global data expected to reach 175 zettabytes by 2025, traditional storage methods face unprecedented challenges, including security risks, limited durability, and high maintenance costs associated with centralized infrastructure. While DNA-based storage systems have demonstrated high density and chemical stability, most existing methods focus primarily on static storage, lacking effective strategies for secure and controllable information transmission. Coli Bond offers a revolutionary approach by combining the molecular precision of DNA storage with the controllable dynamics of synthetic biology, providing an innovative platform for data encryption and storage. In this system, controllable dynamics refer to information transfer regulated by caffeine concentration and temperature. The system leverages synthetic biology to engineer an auxotrophic *Escherichia coli* strain with a caffeine degradation pathway, enabling precise control of information transfer through conditional growth. A temperature-sensitive self-destruction mechanism ensures irreversible destruction of stored information under specific conditions, preventing unauthorized access and enhancing data security. Experimental validation demonstrated the system’s stability and reliability under various real-world conditions, including survival and function in commercial beverages, during transmission cycles, and under temperature variation. The results confirmed high transmission efficiency during initial contact and a rapid decline in strain viability after multiple transfers, providing an inherent layer of security. By integrating the high density of DNA storage with the dynamic control capabilities of synthetic biology, “Coli Bond” offers a secure and adaptable platform for the storage and transmission of DNA-encoded information, paving the way for future advancements in information storage and transmission technologies.

## Introduction

With the rapid global growth of data, information storage and transmission technologies are facing unprecedented challenges. In the field of informatics, existing technologies such as Cloud Storage, Network-Attached Storage (NAS), and Storage Area Networks (SANs) have played significant roles in data management and sharing [1–3]. However, these technologies exhibit notable limitations in terms of security, durability, and sustainability [4,5]. For example, Cloud storage relies on stable network connections and the continuous maintenance of data centers, making it vulnerable to threats such as cyberattacks and data breaches [6,7]. While NAS and SAN have shown improvements in transmission efficiency, their limitations in scalability, cost-effectiveness, and system design restrict their applicability [8]. Furthermore, the risk of data breaches in distributed learning systems has raised widespread concern in recent years [9]. Research has demonstrated that private training data can be maliciously recovered through gradient-sharing mechanisms. In the field of supply chain management, although blockchain technology has enhanced the transparency of data sharing, the risk of information leakage persists [10]. In scenarios requiring strict data confidentiality, such as sensitive commercial information storage, these limitations are particularly pronounced, as traditional technologies struggle to provide robust security. [7,11].

In recent years, DNA-based storage and transmission technologies have rapidly emerged as a research focus in the field of informatics owing to their exceptional molecular properties and information-carrying capacity [12–14]. The high storage density of DNA enables the storage of vast amounts of information in extremely small volumes. Theoretically, one gram of DNA can store approximately 215 petabytes (PBs) of data, and its chemical stability ensures data integrity for decades, even under extreme conditions [15,16]. Moreover, compared with traditional storage media, DNA does not rely on electronic devices, which results in lower energy consumption and longer preservation periods, making it highly significant for long-term archiving and rare data storage [17–19]. In terms of encoding strategies, innovations in DNA storage technology continue to drive improvements in performance. Research has shown that by introducing degenerate bases and optimizing the encoding character set, the information capacity of DNA can be increased to 3.37 bits per character while simultaneously reducing both synthesis and reading costs [20]. Along with the optimization of encoding strategies, advancements in de novo DNA synthesis technology have not only increased storage efficiency but also significantly lowered cost barriers, opening new possibilities for large-scale applications [21].

Sensitive commercial data, such as intellectual property and financial records, depend heavily on cloud storage and encrypted databases. However, these methods remain vulnerable to cyberattacks, insider threats, and long-term degradation [22]. Additionally, advances in quantum computing pose a future risk to encryption security, whereas centralized data centers introduce concerns about physical damage and high maintenance costs [23]. DNA storage offers a durable, secure, and high-density alternative [24]. DNA retains chemical stability for thousands of years within biological environments and is inherently immune to hardware-related degradation. Unlike traditional digital storage, DNA enables biologically secure and decentralized data preservation, making unauthorized access significantly harder [14,25]. With these advantages, DNA storage provides a future-proof solution for businesses requiring long-term, highly secure data protection.

However, DNA storage and transmission technologies still face numerous challenges, including issues related to the reliability and environmental robustness of information transfer, the lack of dynamic system control capabilities, and the limited adaptability of existing technologies in complex scenarios [26]. First, the majority of current research focuses on the synthesis, storage, and reading capabilities of DNA, with relatively limited exploration into its potential for secure information transmission [27–30]. In practical applications, the information transfer process is susceptible to environmental factors, posing risks of data loss or leakage [15,17]. Moreover, even during storage, DNA molecules are prone to degradation through mechanisms such as strand breakage, particularly at room temperature, which may cause irreversible information loss. Moreover, there is currently a lack of effective methods to ensure the precision and confidentiality of information transmission. Existing approaches struggle to achieve high-precision dynamic control during the information storage and transmission process. This is particularly challenging in complex environments, where achieving reliable and controllable information transfer remains an unresolved technical problem. DNA storage technology has demonstrated unique advantages in long-term data archiving because of its high confidentiality and storage density. In previous studies [17–19], existing solutions lack real-time control over the storage and transmission processes. This limitation undermines its performance in terms of both security and efficiency, thereby restricting its widespread application in dynamic scenarios.

Current information storage and transmission systems face a range of limitations across digital and molecular platforms. Cloud-based and centralized systems such as NAS and SAN offer scalable data access but rely heavily on stable networks and data centers, making them prone to cyberattacks, high energy consumption, and limited sustainability. Distributed frameworks, including federated learning and blockchain storage, improve data transparency but still face privacy challenges such as gradient leakage and information reconstruction. DNA-based systems, while promising ultra-high storage density and long-term chemical stability, are mostly implemented in vitro and suffer from strand breakage, lack of dynamic access control, and poor environmental robustness. These limitations highlight the need for a new biological approach that combines secure data retention with externally controllable information transmission (Table 1).

**Table 1:** Comparison of mainstream information storage and transmission systems. This table summarizes the key characteristics, advantages, and limitations of three representative categories: centralized digital storage (Cloud/ NAS/ SAN), distributed data systems (e.g., federated learning and blockchain), and in vitro DNA-based storage.

System Type	Cloud/ NAS/ SAN	Distributed Data Systems	In vitro DNA Storage
**Technology & Features**	Centralized storage with network access	Federated learning, blockchain, shared ledger storage	Synthetic oligos stored in solution or dried formats
**Advantages**	Fast access, scalable, widely adopted	Improved transparency and decentralization	Ultra-high density, chemical stability
**Limitations**	Vulnerable to cyberattacks, energy-intensive, high maintenance	Risk of gradient leakage, partial anonymity, lacks full privacy guarantees	No access control, environmentally fragile, prone to strand breakage

To address these challenges, we developed Coli Bond, a system that integrates DNA storage with synthetic biology to achieve externally regulated access and reliable information transmission. By embedding data in metabolically engineered E. coli, Coli Bond supports caffeine-dependent decoding and temperature-triggered self-destruction, enabling precise control over data flow and lifespan.

Its key features include:

- Metabolism-dependent controlled transfer: Enables real-time, selective propagation via caffeine-dependent growth [31,32].
- Temperature-triggered self-destruction: Irreversibly destroys stored information upon thermal activation [33].
- Dual-layer security: Combines metabolic and temperature control to safeguard access.
- Environmental robustness: Maintains function across real-world conditions such as beverage exposure and sequential transfers.
- Programmable storage lifespan: Leverages the long-term stability of living cells while enabling controlled erasure when needed.

**Fig 1:**
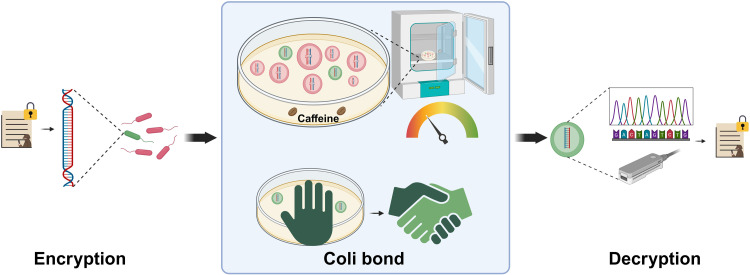
Conceptual diagram of the Coli Bond system.

Combining DNA’s storage potential with dynamic biological control, Coli Bond provides a secure, adaptable platform for next-generation data transmission, with promising applications in complex and decentralized environments (Fig 1).

This diagram illustrates the process of data encryption, storage, transmission, and decryption using the Coli Bond system. Data are first encoded into DNA, utilizing existing or future DNA-based storage methods. The Coli Bond system then encrypts and stores this information, enabling controlled transmission through a caffeine-dependent mechanism and a temperature-sensitive self-destruction feature. Finally, the stored DNA reaches the recipient, where it is decrypted to recover the original data, completing the secure transfer process.

## Materials and methods

### Chemical reagents and medium preparation

In this study, 7-MX (purity 97%) was purchased from Aladdin Biotech Co., Ltd. (Shanghai, China), and xanthine (purity 99%) was purchased from McLean Biochemical Technology Co., Ltd. (Shanghai, China). Caffeine was sourced from guarana extract (containing approximately 22% caffeine). The standard curve for caffeine was constructed using an indirect method involving 7-MX and theobromine. All other chemical reagents used in the experiments were routine molecular biology reagents of analytical grade and were purchased from reputable suppliers.

### Strain construction

The bacterial strains used in this study included *Escherichia coli* (*E. coli*) DH5α and BW25113, purchased from Weidi Biotechnology Co., Ltd. (Shanghai, China). The gene knockout strain BW-ΔguaB was constructed by using the RED recombination method [34–36] to knock out the *guaB* gene and replace it with a kanamycin resistance gene. (S1 Fig) The competent cells were prepared in the laboratory, while the other strains were purchased from Weidi Biotechnology Co., Ltd. (Shanghai, China).

### Plasmid construction and optimization

The plasmid construction in this study combined the Gibson assembly method and Golden Gate ligation method. The Gibson assembly method was used for seamless connection of larger fragments, ensuring efficient and seamless ligation, whereas the Golden Gate method utilized BsaI restriction enzyme digestion and T4 DNA ligase for directional insertion of fragments, which is particularly useful for multifragment constructions. After construction, the plasmids were transformed into competent *E. coli* DH5α cells, which were subsequently plated on LB agar plates containing 50 mg/L antibiotics. The plates were incubated at 37 °C for 12 hours.

Subsequently, positive clones were selected by colony PCR, and the plasmids were extracted and verified by sequencing. The successfully verified plasmid was then transformed into *E. coli* BW25113, resulting in the final engineered strains required for this study.

### Protein expression and screening

Protein expression in engineered strains was performed using the standardized ZYM5052 media. The expression was induced in medium containing arabinose (0.2%) and isopropyl-β-D-thiogalactopyranoside (IPTG). The cultures were incubated at 37 °C with shaking at 200 rpm for 12–16 hours. Protein expression levels were verified by SDS‒PAGE, and functional screening was conducted on the expressed products.

### Construction of the coli bond system

The Coli Bond system was constructed by modularly engineering *Escherichia coli* BW25113 to incorporate caffeine-dependent growth control and a conditional self-destruction mechanism. To establish an auxotrophic background, the *guaB* gene was knocked out via λ-Red recombination and replaced with a kanamycin resistance cassette, yielding strain BW-ΔguaB (S1 Fig). Growth assays in M9 minimal medium verified the strain’s dependence on exogenous purines, as it exhibited no growth without xanthine supplementation (S2 Fig).

To convert this dependency from xanthine to caffeine, a caffeine demethylation pathway derived from *Pseudomonas putida* CBB5 was introduced. Genes encoding the N-demethylases (*ndmA*, *ndmB, ndmC*) and associated redox partners (*ndmD*) were assembled into a plasmid using Gibson and Golden Gate cloning strategies and placed under constitutive or inducible promoters. The functionality of the Decaf pathway was confirmed by the restored growth of BW-ΔguaB in M9 medium supplemented with caffeine as the sole purine source (S3 Fig).

For the self-destruction module, the *dpnI* restriction endonuclease gene was cloned under the control of an inducible promoter. Upon activation, DpnI expression led to methylated DNA degradation, resulting in irreversible loss of genomic integrity and cell death.

All plasmids were constructed and verified in *E. coli* DH5α via colony PCR and Sanger sequencing, then transformed into BW-ΔguaB. Engineered strains were cultured in ZYM5052 auto-induction medium supplemented with 0.2% arabinose and 0.1 mM IPTG at 37 °C with shaking at 200 rpm. Target protein expression was confirmed by SDS–PAGE, and functional validation was conducted through caffeine-responsiveness assays and temperature-induced viability tests.

## Results

### Construction of auxotrophic E. coli and the decaf pathway

In this study, we constructed an auxotrophic *E. coli* strain and introduced a caffeine degradation pathway (Decaf pathway) [37,38] to develop a conditional growth system that enhances the security and practicality of information transmission.

**Fig 2:**
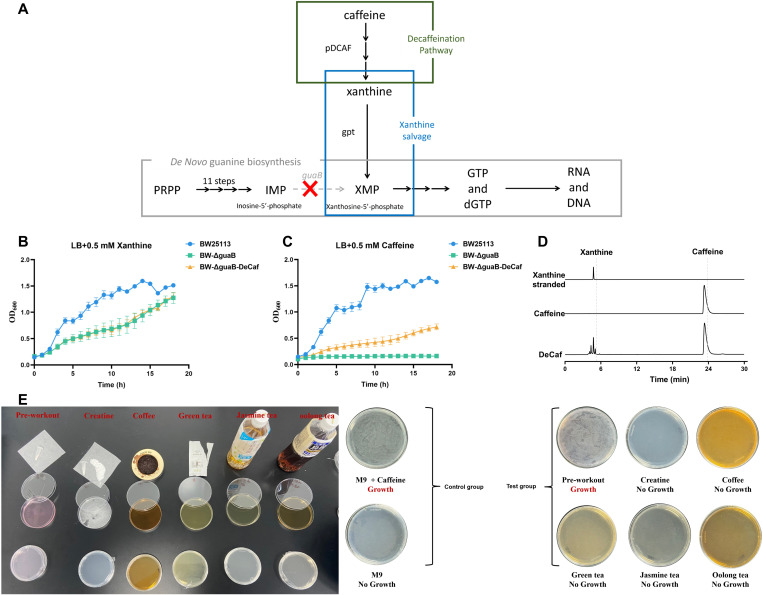
Construction and validation of the Decaf pathway and auxotrophic *E. coli* system. (A) Schematic of the purine biosynthesis pathway and the integration of the Decaf pathway. The auxotrophic strain BW-ΔguaB/Decaf was engineered to metabolize caffeine into xanthine by deleting the *guaB* gene and introducing the Decaf pathway. (B-C) Growth curves demonstrating system functionality: growth restoration of BW-ΔguaB in LB medium supplemented with xanthine (B) and caffeine utilization by BW-ΔguaB/Decaf in LB medium (C). (D) Verification of caffeine conversion to xanthine via the Decaf pathway. The figure shows three HPLC chromatograms, with the signals from top to bottom corresponding to xanthine, caffeine, and the results following Decaf pathway treatment (DeCaf), respectively. These results demonstrate that the Decaf pathway effectively converts caffeine into xanthine. (E) Growth of BW-ΔguaB/Decaf in commercially available caffeine-containing beverages, illustrating its potential for real-world applications.

By knocking out the *guaB* gene, we successfully created the auxotrophic strain BW-ΔguaB, which depends on exogenous xanthine or its derivatives for growth. The *guaB* gene encodes a key enzyme in the de novo guanine biosynthesis pathway, and its deletion disrupts the production of xanthine-5’-phosphate, a crucial intermediate [39] (Fig 2A). As a result, the BW-ΔguaB strain is unable to grow under conditions lacking xanthine (S2 Fig). However, the growth of BW-ΔguaB could be restored by supplementing the medium with 0.5 mM xanthine, confirming its dependency on exogenous xanthine and validating the successful construction of the auxotrophic host (Fig 2B).

In the context of information decoding, caffeine is more readily available and cost-effective than xanthine. Thus, the use of caffeine enhances the security and practicality of the system. To enable the strain to utilize caffeine as a precursor for xanthine, we integrated the key caffeine degradation genes from *Pseudomonas putida* CBB5 into the BW-ΔguaB strain, constructing the engineered strain BW-ΔguaB/Decaf, thereby allowing the strain to rely on caffeine rather than exogenous xanthine (Fig 2A and S3 Fig). Next, to verify whether the Decaf pathway could successfully convert caffeine to xanthine, we performed HPLC analysis. The HPLC chromatograms show signals corresponding to xanthine, caffeine, and Decaf pathway-treated samples (DeCaf) from top to bottom. These results indicate that the Decaf pathway effectively converts caffeine into xanthine (Fig 2D). Functional analysis further confirmed that the engineered strain BW-ΔguaB/Decaf could grow normally in caffeine-containing media, demonstrating that the Decaf pathway successfully enabled the strain to metabolize caffeine into xanthine, thereby supporting guanine synthesis (Fig 2C).

Further experiments verified that BW-ΔguaB/Decaf could grow stably in various commercially available caffeinated beverages, such as energy drinks, coffee, and preworkout mixtures (Fig 2E) [40].

### Self-destruction mechanism verification

To ensure the security of encoded information and prevent unauthorized access, we designed a temperature-controlled self-destruction system. This system triggers the degradation of genomic DNA carrying information and induces cell death, thereby resulting in irreversible data destruction. The core of the system is the temperature-sensitive Y38 promoter, which regulates the expression of the DpnI gene. DpnI encodes a DNA endonuclease that specifically cleaves GATC sequences, which are abundant in the *E. coli* genome. At the commonly used cultivation temperature of 37 °C, the Y38 promoter is activated, inducing DpnI expression, which leads to genome fragmentation and self-destruction of the cell. We exploited this temperature, which is typically considered optimal for bacterial growth, to enable the encrypted strain to destroy the information under standard conditions, thus realizing a unique strategy for confidentiality and destruction.

**Fig 3:**
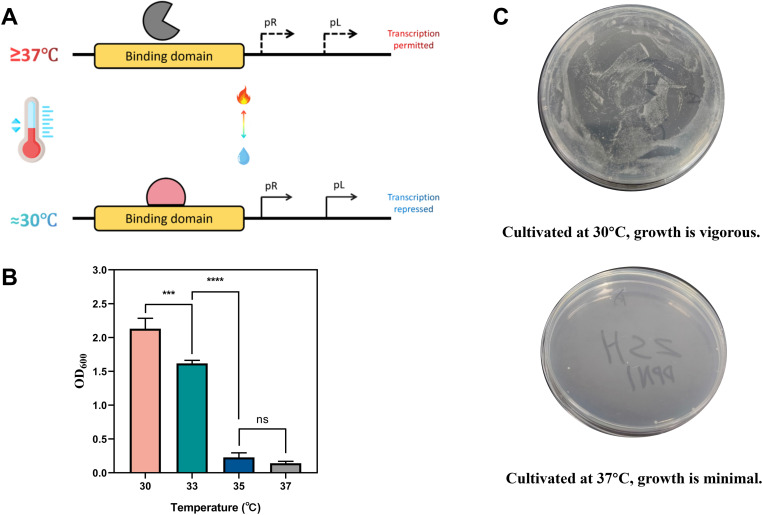
Validation of the temperature-sensitive self-destruction mechanism. (A) Schematic representation of the temperature-sensitive self-destruction system. At 30 °C, the repressor binds to the promoter region, preventing DpnI gene expression. At 37 °C, the repressor dissociates from the promoter, activating DpnI expression, leading to DNA degradation and cell death. (B) Growth analysis of the BW-ΔguaB/Decaf/Y38-DpnI-TrrnB strain at different temperatures. OD600 measurements indicate robust growth at 30 °C, which is slightly reduced at 33 °C (***p < 0.001). Growth is almost entirely inhibited at 35 °C (****p < 0.0001), with no significant difference (ns) between 35 °C and 37 °C, confirming effective self-destruction at higher temperatures. (C) Plate cultivation experiments confirming the temperature-sensitive effect. At 30 °C, the strain grows vigorously, whereas at 37 °C, growth is markedly reduced, further demonstrating the efficacy of the self-destruction mechanism.

During the construction process, we assembled the Y38 promoter (1488 bp), the DpnI gene (805 bp), and the TrrnB terminator into the PY38a plasmid via the Gibson assembly method, generating the composite part Y38-DpnI-TrrnB (BBa_K5480009). At low temperatures (30 °C), the lambda repressor (cI dimer) binds to and inhibits the promoter, preventing DpnI expression and maintaining genomic stability. Upon temperature elevation to 37 °C, the cI dimer dissociates, activating the promoter and driving DpnI expression, thus initiating the self-destruction process (Fig 3A).

The temperature sensitivity of this system was first validated in *E. coli* BW25113 cells. At 30 °C, the cells harboring the Y38-DpnI-TrrnB construct grew normally, whereas at 37 °C, growth was almost completely inhibited (Fig 3C). The OD600 values further validated the temperature-sensitive characteristics of the system. The results revealed that the strain exhibited robust growth at 30 °C, significantly reduced growth at 33 °C (***p < 0.001), and was nearly completely inhibited at 35 °C (****p < 0.0001), with no significant difference (ns) between 35 °C and 37 °C (Fig 3B). These results demonstrate that the self-destructive mechanism can be effectively triggered at the set temperature threshold, enabling the controllable destruction of engineered strains. Moreover, this design prevents strain leakage and misuse after information transmission is completed, further enhancing the security of data storage and transmission.

### Transmission

After validating the conditional growth mechanism and the temperature-controlled self-destruction system, we further investigated the efficacy and limitations of the engineered strain in information transmission. To assess the practical application value of this system in information storage and transfer, we designed a “handshake” experiment to simulate the transmission process and evaluate the survival capacity of the strain after multiple contacts.

**Fig 4:**
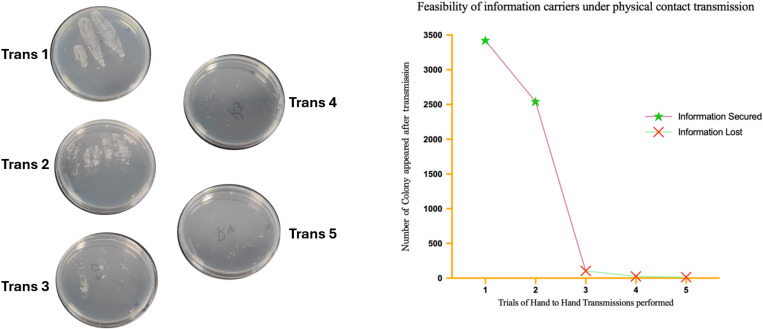
Viability assessment of information carriers during physical contact transmission. The left panel shows plate cultivation results from sequential hand-to-hand transmissions, reflecting changes in strain viability. The right panel shows a marked decline in colony numbers after the third transmission, culminating in complete loss of viability. These findings confirm the system’s reliability in initial transmissions and enhanced security in preventing unauthorized multiple transfers.

In the experiment, five glove-wearing volunteers participated sequentially. The first volunteer applied a bacterial suspension containing the BW-ΔguaB/Decaf/Y38-DpnI-TrrnB strain onto their hands, which was then transferred to the next volunteer’s hands, and so on. By measuring the colony count after each transmission, we observed a gradual decline in the strain’s survival rate. In the first and second transfers, the colony counts were relatively high, whereas in the third, fourth, and fifth transfers, the survival rate significantly decreased (Fig 4). This transmission decay phenomenon highlights the unique advantages of biological carriers in terms of information security—an entropy-driven security mechanism.

These results indicate that although the engineered strain can effectively facilitate secure information transfer during initial contact, its survival capacity rapidly decreases after multiple transfers. This phenomenon highlights two key features of the dual-functional system: first, the strain’s high efficiency in the initial transmission ensures the reliability and security of information transfer; second, the rapid decline in survival during subsequent transfers provides a natural protective layer to the system, making unauthorized or repeated transmissions to restore the information difficult, thereby enhancing the system’s security and covert nature.

## Discussion

### Key features and implications

The “Coli Bond” system created in this study is an innovative approach that combines synthetic biology with data security. This provides an effective method for secure information storage and sharing using microorganisms. This design incorporates special growth conditions and a temperature-controlled self-destruction mechanism to construct a complex system for protecting stored information.

“Coli Bond” offers an additional solution to biosafety concerns by utilizing the DeCaf pathway, which allows bacterial growth only in the presence of caffeine or its derivatives. This approach not only enhances biosafety but also aligns with the international trend in bioengineering, which involves the design of environmentally dependent mechanisms to control the behavior of organisms. By replacing hypoxanthine with caffeine as a metabolic product, the system’s practicality can be further improved, as caffeine is widely found in everyday consumer products. This finding is consistent with previous research in microbial engineering, where caffeine was converted into valuable metabolites [41,42]. The core advantage of the system lies in its dual-composite mechanism: first, caffeine, a compound widely found in everyday consumer products, enables seamless integration of the decryption key into existing supply chains without the need for specialized chemical distribution channels [43,44]; second, the auxotrophic strain enhances resistance to unauthorized access by strictly controlling its metabolic pathways, limiting its growth to specific conditions, and significantly increasing the difficulty of brute-force cracking. Experimental validation further confirmed the strain’s stable growth in commercial beverages, demonstrating the system’s practical potential: the decryption process requires both specialized biological knowledge, such as the revival of the strain, and precise chemical control, including the regulation of caffeine concentration. This dual-authentication mechanism, by introducing “biochemical uncertainty,” surpasses traditional digital encryption systems in terms of security, providing a more robust safeguard for data storage and transmission.

The temperature-sensitive self-destruction system serves as a backup mechanism to protect sensitive data. Under regular *E. coli* cultivation conditions, the DpnI gene is activated, leading to the degradation of DNA containing the information, rendering it inaccessible or preventing unauthorized use. This concept is similar to the widely accepted gene kill switches, which play a significant role in enhancing the biosafety and controllability of synthetic microbial systems [45]. Moreover, this strategy effectively addresses biosafety concerns related to the deployment of genetically modified organisms (GMOs) in uncontrolled environments [46]. The design of the self-destruction mechanism offers unprecedented security for data storage and transmission, particularly when sensitive data are handled, effectively preventing data theft or tampering. As biological data storage technologies continue to evolve, ensuring absolute confidentiality and the complete destruction of data in the case of unauthorized access has become a critical issue [47]. Our temperature-controlled self-destruction system provides an innovative solution to this challenge and is applicable to future high-security storage fields, ensuring that once information is decrypted or accessed without authorization, it is immediately destroyed, thus preventing data leakage or misuse.

The experimental results show that while the engineered strain effectively facilitates information transfer upon initial contact, its survival capacity rapidly declines after multiple transfers, highlighting two key features of the dual-functional system: first, the strain’s high efficiency ensures the reliability and security of the initial transmission; second, the rapid decline in survival during subsequent transfers provides a natural protective barrier, preventing unauthorized or repeated transmissions from restoring information, further enhancing the system’s security and covert nature. This transmission decay phenomenon reveals the unique advantages of biological carriers in terms of information security—an entropy-driven security mechanism. The entropy-driven security mechanism is based on the randomness and irreversibility of biological systems, making unauthorized access more difficult to achieve. Each physical contact induces mechanical damage and nutrient depletion, creating a natural “biological one-time password (OTP)” system, turning biological limitations into security advantages [48]. In practical applications, unauthorized intermediaries need laboratory-level environmental control capabilities and real-time monitoring technologies to exceed the limits of three effective transfers, thus creating an insurmountable technical barrier.

### Challenges and future directions

However, the system also has inherent limitations. The use of antibiotic resistance genes as selection markers may pose potential biosafety risks, including the horizontal transfer of resistance traits to native microbial populations [49]. How to ensure the isolation of genetically modified organisms in the natural environment and how to effectively avoid their potential harm to local ecosystems will be issues that cannot be ignored in future research [50–52]. The literature has proposed antibiotic-free metabolic complementation screening methods [53], which should be considered in future iterations of this system. For example, the use of caffeine toxicity as a screening tool for target strains could provide an effective alternative.

Although the “Coli Bond” system enhances data security through the “biological one-time password” mechanism, once the information is successfully destroyed, the recovery of the data becomes extremely difficult. This irrecoverability may present challenges for privacy protection in some scenarios. For example, in the case of misoperation or accidental damage, the data may never be restored, causing irrevocable losses to users. Therefore, how to provide a certain data recovery capability while ensuring data security to address unexpected situations is also one of the directions of future improvement [54].

DNA holds significant potential for information storage, and improvements in DNA synthesis and reading technologies have enabled the long-term, large-scale storage of information. Tools such as portable sequencers (e.g., nanopore technology) are continuously being developed to address real-world challenges in DNA synthesis and reading. These advancements are crucial to enhancing the practicality and scalability of DNA-based data storage systems [55–57].

To further improve the practicality and scalability of the “Coli Bond” system, interdisciplinary collaboration and technical integration play crucial roles. In addition to the basic research of synthetic biology, the research and development of data storage systems also needs to be closely coordinated with computer science, information technology, materials science and other fields [58,59]. For example, artificial intelligence can be used for data encryption and decoding, while nanotechnology may optimize the physical storage media. [60–62] Moreover, incorporating mathematical modeling and system-level simulation could enhance the quantitative understanding of signal-controlled growth and improve the fidelity of data transmission. These interdisciplinary approaches are expected to unlock more innovative applications and technological breakthroughs for the *Coli Bond* platform.

## Conclusion

In this study, we aimed to develop a secure information storage and transmission platform by integrating synthetic biology with DNA-based technologies. To achieve this, we developed Coli Bond, a dual-function encryption system based on synthetic biology for secure information storage and transmission. This system integrates metabolism-dependent growth control and a temperature-sensitive self-destruction mechanism. By knocking out the guaB gene and introducing the Decaf (caffeine degradation) pathway, we engineered an *E. coli* strain that requires exogenous caffeine for growth, thereby enabling information decoding control. Additionally, a temperature-sensitive DpnI restriction enzyme system was designed to trigger irreversible DNA degradation at 37 °C, ensuring data security. The experimental results demonstrated that the system reliably preserves information integrity and ensures security during storage and transmission. The engineered strain showed high survival rates during initial transmission but experienced a rapid decline after multiple transfers, enhancing data security. Furthermore, the temperature-controlled self-destruction mechanism effectively triggered cell lysis, preventing unauthorized access. In addition, the Coli Bond system holds significant potential for applications in commercial confidential data storage and transmission, steganographic communication, personal privacy protection, and DNA-based information computing. Although the Coli Bond system demonstrates strong potential as a biological encryption strategy, several limitations remain. First, the current reliance on antibiotic resistance markers poses potential biosafety risks due to the possibility of horizontal gene transfer in environmental settings. Second, the methods for encoding information into bacteria and retrieving information from bacteria require further improvement. Future enhancements could include adopting antibiotic-free selection strategies, integrating CRISPR-based control systems such as CRISPR-Cas9 for targeted data insertion, CRISPRa/i for programmable regulation of information accessibility, and CRISPR-Cas3 for controlled sequence deletion, and incorporating nanopore sequencing technologies to enhance overall biosafety and operational efficiency [63–66].This study presents a novel approach to bioencryption and secure data transmission, with potential applications in confidential information storage, steganography, and DNA-based computation. In conclusion, Coli Bond not only advances the frontier of DNA-based storage but also opens new possibilities for secure communication in an era where data privacy is paramount.

## Supporting information

S1 Fig RED Homologous Recombination Gene Knockout in *E. coli.* This figure illustrates the process of gene knockout using the RED (Recombination Engineering) system, a highly efficient genomic editing technology based on the λ phage Red system. The system includes three key enzymes: Exo (5’ → 3’ exonuclease), Beta (single-stranded DNA binding protein), and Gam (antinuclease protein), which facilitate homologous integration or replacement of foreign DNA into the host genome [35,36,67,68]. In this study, the upstream and downstream fragments of the *guaB* gene were amplified by PCR, along with the kanamycin resistance gene. These fragments were assembled into the linearized pccdK2 vector using the Gibson assembly method to generate a targeting fragment, which was then transformed into competent *E. coli* DH5α cells. Positive clones were selected by colony PCR and verified by sequencing. The verified plasmid was transformed into *E. coli* BW25113 [69], resulting in the successful construction of the gene knockout strain BW-ΔguaB (S2 Fig).
(TIF)

S2 Fig Growth of *E. coli* BW-ΔguaB in M9 medium. (A) Growth of the auxotrophic *E. coli* strain BW-ΔguaB in M9 medium supplemented with 0.5 mM xanthine. (B) Growth of the strain in M9 medium without any added xanthine. The strain shows a clear dependency on the supplementation of xanthine for growth, as evidenced by the lack of growth in the medium without xanthine.
(TIF)

S3 Fig Construction of a plasmid for caffeine conversion to xanthine. This figure illustrates the plasmid constructed by incorporating caffeine demethylation genes derived from *Pseudomonas putida* CBB5. The positions of promoters, ribosome binding sites, and terminators are indicated. The purpose of this pathway is to convert caffeine into xanthine, thereby supplementing the xanthine required by the BW-ΔguaB strain and shifting its dependence from xanthine to caffeine.
(TIF)

S4 File Supplementary Data. This zip file contains the raw data sets supporting the findings of this study.
(ZIP)
