# Supplementary figures and images for: Coli bond: A dual-function encryption system for secure information storage and transmission by microorganisms
Source: PLoS One. 2025 Jun 11;20(6):e0325926. doi: 10.1371/journal.pone.0325926 (PMC12157041; doi:10.1371/journal.pone.0325926)

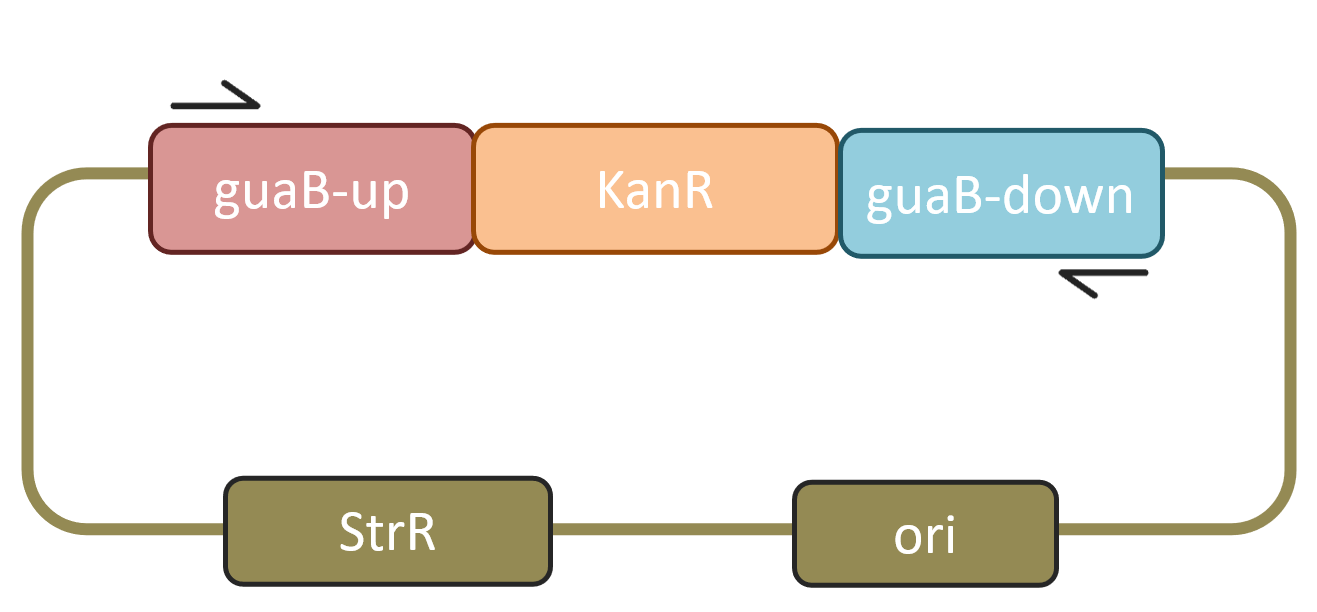

Supplement: S1 Fig — This figure illustrates the process of gene knockout using the RED (Recombination Engineering) system, a highly efficient genomic editing technology based on the λ phage Red system. The system includes three key enzymes: Exo (5’ → 3’ exonuclease), Beta (single-stranded DNA binding protein), and Gam (antinuclease protein), which facilitate homologous integration or replacement of foreign DNA into the host genome [35,36,67,68]. In this study, the upstream and downstream fragments of the guaB gene were amplified by PCR, along with the kanamycin resistance gene. These fragments were assembled into the linearized pccdK2 vector using the Gibson assembly method to generate a targeting fragment, which was then transformed into competent E. coli DH5α cells. Positive clones were selected by colony PCR and verified by sequencing. The verified plasmid was transformed into E. coli BW25113 [69], resulting in the successful construction of the gene knockout strain BW-ΔguaB (S2 Fig). (TIF) [file pone.0325926.s001.tif]

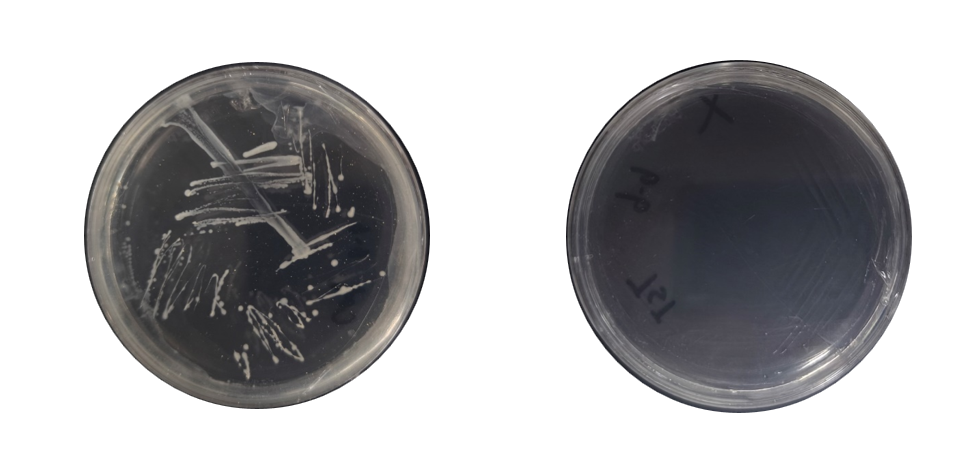

Supplement: S2 Fig — (A) Growth of the auxotrophic E. coli strain BW-ΔguaB in M9 medium supplemented with 0.5 mM xanthine. (B) Growth of the strain in M9 medium without any added xanthine. The strain shows a clear dependency on the supplementation of xanthine for growth, as evidenced by the lack of growth in the medium without xanthine. (TIF) [file pone.0325926.s002.tif]

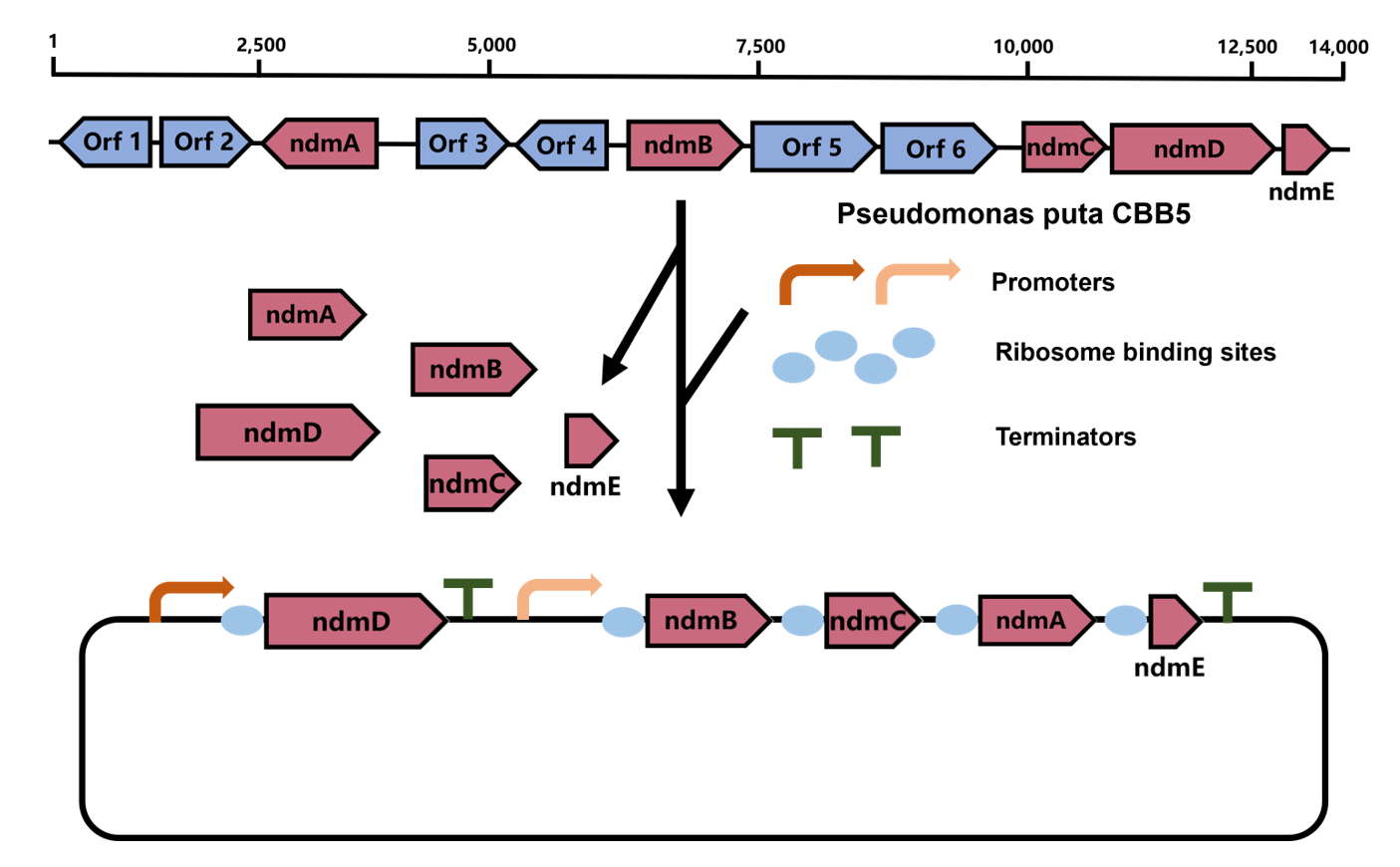

Supplement: S3 Fig — This figure illustrates the plasmid constructed by incorporating caffeine demethylation genes derived from Pseudomonas putida CBB5. The positions of promoters, ribosome binding sites, and terminators are indicated. The purpose of this pathway is to convert caffeine into xanthine, thereby supplementing the xanthine required by the BW-ΔguaB strain and shifting its dependence from xanthine to caffeine. (TIF) [file pone.0325926.s003.tif]
